# Perfil Aterosclerótico da Artéria Carótida como Preditor de Risco para Reestenose após Implante de *Stent* Coronário

**DOI:** 10.36660/abc.20190650

**Published:** 2021-04-08

**Authors:** Cássia da Silva Antico Rodrigues, Rodrigo Bazan, Fabrício Moreira Reis, Caroline F. S. Mazeto Pupo da Silveira, Lívia Maria Severino Hueb, Fábio Cardoso de Carvalho, Hélio Rubens de Carvalho Nunes, Katashi Okoshi, João Carlos Hueb, Silméia Garcia Zanati Bazan

**Affiliations:** 1 Universidade Estadual Paulista Julio de Mesquita Filho Faculdade de Medicina Campus de Botucatu BotucatuSP Brasil Universidade Estadual Paulista Julio de Mesquita Filho - Faculdade de Medicina Campus de Botucatu, Botucatu, SP – Brasil

**Keywords:** Doença Arterial Coronariana, Aterosclerose, Reestenose Coronária, Stents, Angioplastia Coronária com Balão, Artérias Carótidas/ultrassonografia, Placa Aterosclerótica

## Abstract

**Fundamento:**

A incidência de reestenose da artéria coronária após o implante de um *stent* não farmacológico é mais baixa que na angioplastia com balão; no entanto, ainda apresenta altas taxas.

**Objetivo:**

O objetivo deste estudo foi identificar novos indicadores de risco para reestenose de *stent* usando ultrassonografia das carótidas que, em conjunto com indicadores já existentes, ajudariam na escolha do *stent*.

**Métodos:**

Realizamos um estudo prospectivo transversal incluindo 121 pacientes consecutivos com doença arterial coronariana que foram submetidos à intervenção coronária percutânea com angiografia nos 12 meses anteriores. Após os casos de reestenose de *stent* serem identificados, os pacientes foram submetidos à ultrassonografia de carótidas para avaliar a espessura da camada íntima média e placas ateroscleróticas. Os dados foram analisados por regressão múltipla de Cox. O nível de significância foi p<0,05.

**Resultados:**

A idade mediana dos pacientes foi de 60 anos (1º quartil = 55, 3º quartil = 68), e 64,5% dos pacientes eram do sexo masculino. A angiografia coronária mostrou que 57 pacientes (47,1%) apresentaram reestenose de *stent*. Cinquenta e cinco pacientes (45,5%) apresentaram placas ateroscleróticas ecolucentes nas artérias carótidas e 54,5% apresentaram placas ecogênicas ou nenhuma placa. Dos pacientes que apresentaram placas ecolucentes, 90,9% apresentaram reestenose do *stent* coronário, e daqueles com placas ecogênicas ou nenhuma placa, 10,6% apresentaram reestenose de *stent*. A presença de placas ecolucentes nas artérias carótidas aumentou o risco de reestenose de *stent* coronário em 8,21 vezes (RR=8,21;IC95%: 3,58-18,82; p<0,001).

**Conclusões:**

A presença de placas ateroscleróticas ecolucentes na artéria carótida constitui um preditor de risco de reestenose de *stent* coronário e deve ser considerada na escolha do tipo de *stent*a ser usado na angioplastia coronária.

## Introdução

O desenvolvimento de *stents* não farmacológicos (SNF) foi um avanço importante na angioplastia com balão para o tratamento de doença arterial coronariana sintomática. Com o uso de *stents*, pode-se evitar a reestenose por meio da atenuação da retração elástica e remodelamento geométrico negativo, resultando na redução do lúmen do vaso.^1^ Contudo, a necessidade de novas revascularizações devido a reestenoses de *stent* ainda era alta, ocorrendo em 10-20% dos pacientes, causadas principalmente por crescimento excessivo da neoíntima, muitas vezes superior à hiperplasia intimal observada na angioplastia simples com balão.^2^^,^^3^

Mais recentemente, os *stents* farmacológicos (SF) foram desenvolvidos para reduzir a elevada taxa de reestenose observada com os SNF e a necessidade de revascularização. Ensaios clínicos confirmaram uma redução de 50% a 70% na necessidade de revascularizações da lesão alvo com o uso de SF em comparação a SNF, apesar de não ter sido observada diferença significativa na taxa de mortalidade global entre eles.^4^^-^^9^ Esses resultados levaram à recomendação do uso de SF na intervenção coronária percutânea. Porém, esses *stents* são de alto custo e exigem um longo período de terapia antiplaquetária dupla para prevenir a ocorrência de trombose, não sendo recomendados para todos os pacientes.^10^

Em algumas situações, tais como diabetes mellitus, envolvimento de pequenos vasos, *stent* dentro de outro *stent*, lesões de bifurcação, lesões extensas ou múltiplas, e enxerto de veia safena, a angioplastia com implante de *stent* apresenta alto risco de reestenose (30-60%). Nessas condições, os SF são mais indicados.^11^

Além das situações mencionadas, pouco se sabe sobre a importância das placas ateroscleróticas nas artérias carótidas e sua correlação com reestenose de *stent*.^12^

Tal correlação é possível, uma vez que inflamação é comum em ambos os casos. Segundo Corrado et al.,^13^ em pacientes submetidos ao implante de *stent* coronário, observa-se uma maior frequência de reestenose de *stent* em pacientes com maior espessura da camada íntima-média (EIM) da parede da carótida e mais placas ateroscleróticas nas artérias carótidas.

Uma vez que a maioria dos indicadores de risco para reestenose de *stent* refere-se a aspectos angiográficos, o objetivo do estudo foi correlacionar, usando-se ultrassonografia, o perfil aterosclerótico da artéria carótida com reestenose de *stent* coronário, com ênfase na presença de placas ecolucentes.

## Pacientes e métodos

### Pacientes

O estudo foi aprovado pelo comitê de ética da Faculdade de Medicina de Botucatu. Todos os pacientes assinaram o termo de consentimento antes de participarem do estudo. Conduzimos um estudo prospectivo transversal incluindo 121 pacientes consecutivos com doença arterial coronariana crônica entre fevereiro e dezembro de 2015. Todos os pacientes haviam sido submetidos à intervenção coronária percutânea e outra angiografia dentro de 12 meses. As angiografias foram indicadas para estratificação de risco de angina, ou realizadas após confirmação de isquemia miocárdica em testes “provocativos” (teste de esforço com exercício ou cintilografia do miocárdio com estresse). Com base na entrevista direta, identificamos quais pacientes apresentaram diabetes mellitus, dislipidemia ou hipertensão arterial. Também identificamos se os pacientes eram fumantes, e quais medicamentos usavam. A angiografia coronária detectou implantação de *stent* prévia nas artérias coronárias (artéria coronária direita, artéria coronária circunflexa, artéria coronária descendente anterior, e seus respectivos ramos).

### Ultrassom com Doppler das Carótidas

Todos os exames foram realizados por um ultrassonografista usando um aparelho Vivid S6 (General Electric Medical Systems, Tirat Carmel, Israel) equipado com um transdutor linear de frequência 8MHz. O ultrassom da artéria carótida com Doppler foi realizado com o paciente na posição supina. As imagens da carótida, incluindo espessura da íntima-média foram analisadas conforme determinado nos consensos da Sociedade Americana de Ecocardiografia (*American Society of Echocardiography*)^14^ e de Mannheim,^15^ e registradas e armazenadas em um CD. A classificação da EIM por sexo e idade foi feita com base nos valores do percentil 75 propostos no estudo CAPS.^16^

A EIM foi medida utilizando-se um padrão de linha dupla visualizado por ecotomografia em ambas as artérias carótidas comuns em uma imagem longitudinal. Esse padrão de linha dupla corresponde às extremidades das interfaces lúmen–íntima e média–adventícia. Os valores médios foram calculados em um segmento de 10 mm próximo à parede posterior do bulbo carotídeo. A placa foi considerada quando uma estrutura focal invadisse o lúmen arterial em pelo menos 0,5mm, ou correspondesse a 50% do valor da EIM circundante, ou quando a medida da espessura fosse maior que 1,5 mm, medida desde a interface média-adventícia à interface lúmen-íntima. As placas foram descritas segundo classificação de Gray-Weale et al.,^17^ Em resumo, placas tipo 1 são aquelas uniformemente ecolucentes; placas tipo II são predominantemente ecolucentes; placas tipo III são placas predominantemente ecogênicas; e as placas tipo IV são uniformemente ecogênicas. Para a análise estatística, os tipos I e II foram chamadas ecolucentes e as tipo III e IV ecogênicas.

### Angiografia coronária

As angiografias coronárias foram realizadas por cateterismo cardíaco transradial. Após angiografia coronária seletiva e identificação do *stent*, a reestenose foi avaliada por angiografia quantitativa. Reestenose de *stent* foi definida como redução do lúmen de 50% ou mais.^18^^,^^19^

### Análise estatística

As variáveis contínuas foram apresentadas como medianas e valores mínimos e máximos. As variáveis categóricas foram expressas como valores absolutos ou frequência (%). A análise de preditores de risco para reestenose de *stent* em 12 meses de seguimento foi realizada em duas etapas. Na etapa 1, foi estimado o risco relativo individual para cada preditor potencial; na fase 2, o modelo de regressão múltipla de Cox foi ajustado para o risco de reestenose de *stent* com os preditores mais fortemente associados (p<0,05) com reestenose detectados na fase 1. Valores de p<0,05 foram considerados estatisticamente significantes. Todas as análises estatísticas foram realizadas usando o SPSS v21.0.

## Resultados

A idade mediana dos 121 pacientes foi 60 anos (1º quartil = 55; 3º quartil = 68); 78 pacientes (64,5%) eram do sexo masculino. Cinquenta e oito (47,9%) dos pacientes eram fumantes, 47 (38,8%) diabéticos, 91 (75,2%) apresentaram hipertensão sistêmica, e 119 (98,3%) apresentaram dislipidemia. Após ajuste do modelo de regressão múltipla de Cox quanto ao risco de reestenose de *stent* por potenciais preditores, observamos que não houve diferença estatisticamente significante na distribuição dessas variáveis nos subgrupos com ou sem reestenose de *stent* (Tabela 1).

**Tabela 1:** Risco de reestenose de stent estimado para cada variável

Variável		RR	IC95%	p
Idade		0,99	0,96-1,02	0,555
Sexo masculino		1,87	1,01-3,46	0,048
**História clínica**				
	HA	0,85	0,47-1,51	0,567
	DM	1,07	0,63-1,81	0,815
	Tabagismo	1,21	0,72-2,03	0,478
	Dislipidemia	0,94	0,13-6,80	0,952
**US artérias carótidas**				
	Placas ecolucentes	8,57	3,89-18,90	<0,001
	EIM (aumentada)	1,88	1,11-3,15	0,017
**Coronária com stent**				
	ADA	1,23	0,72-2,07	0,450
	ACD	0,76	0,44-1,32	0,330
	ACE	0,85	0,45-1,60	0,607

HA: hipertensão arterial; DM: diabetes mellitus; US: ultrassonografia; EIM: espessura da camada íntima-média; ADA: artéria descendente anterior esquerda; ACD: artéria coronária direita; ACE: artéria circunflexa esquerda

Os locais de *stent* foram: artéria descendente anterior esquerda (ADA) em 50 pacientes (41,3%); artéria coronária direita (ACD) em 34 pacientes (28,1%); artéria circunflexa esquerda (ACE) em 19 pacientes (15,7%), ADA e ACD em 9 pacientes (7,4%), ADA e ACE em 5 pacientes (4,1%), e ACD e ACE em 4 pacientes (3,3%). As angiografias mostraram que 57 pacientes (47,1%) apresentaram reestenose de *stent* coronário, e o local do *stent* não influenciou na taxa de reestenose de *stent* (Tabela 1).

A maioria dos pacientes tomava aspirina (97,5%), estatina (92,6%), inibidores da enzima conversora de angiotensina ou bloqueadores de receptor de angiotensina (80,2%), betabloqueadores (88,4%), e 27,3% dos pacientes usavam clopidogrel.

**Figura 1:**
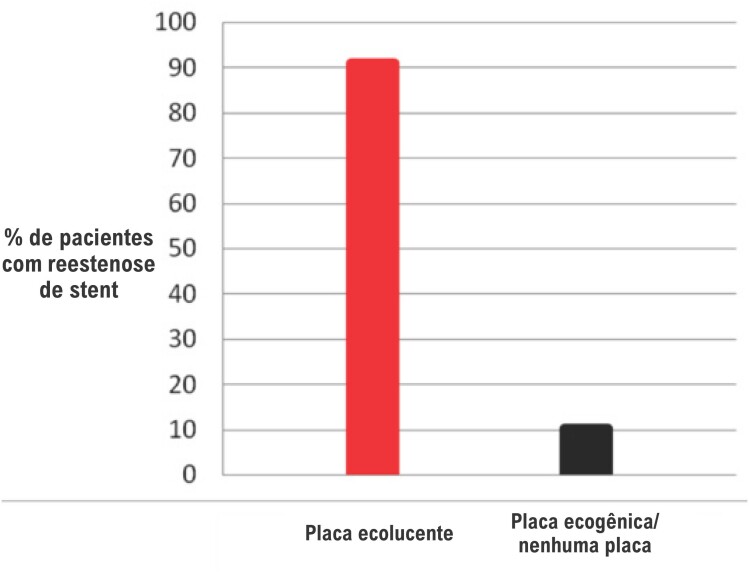
Porcentagem de pacientes com reestenose de stent coronário nos grupos de pacientes com placa ecolucente e pacientes com placas ecogênicas ou nenhuma placa nas artérias carótidas.

Cinquenta e cinco pacientes (45,5%) dos pacientes apresentaram placas ecolucentes nas artérias carótidas e 66 pacientes (54,5%) apresentaram placas ecogênicas ou nenhuma placa. Cinquenta (90,9%) pacientes com placas ecolucentes, e somente sete (10,6%) daqueles com placas ecogênicas ou nenhuma placa apresentaram reestenose de *stent* (Figura 1).

**Figura 2:**
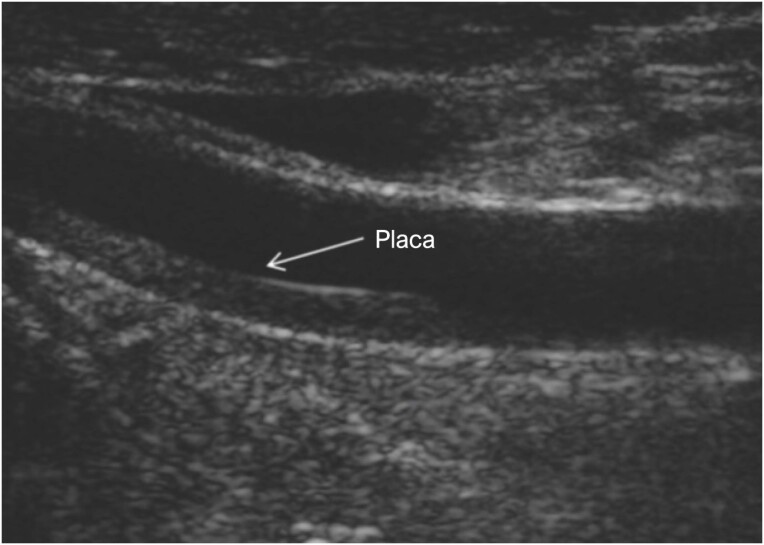
Imagens ultrassonográfias de placas ateroscleróticas tipo II na artéria carótida esquerda.

As imagens de ultrassonografia das placas carótidas e os achados angiográficos das artérias coronárias são apresentadas nas Figuras 2 e 3, respectivamente.

**Figura 3:**
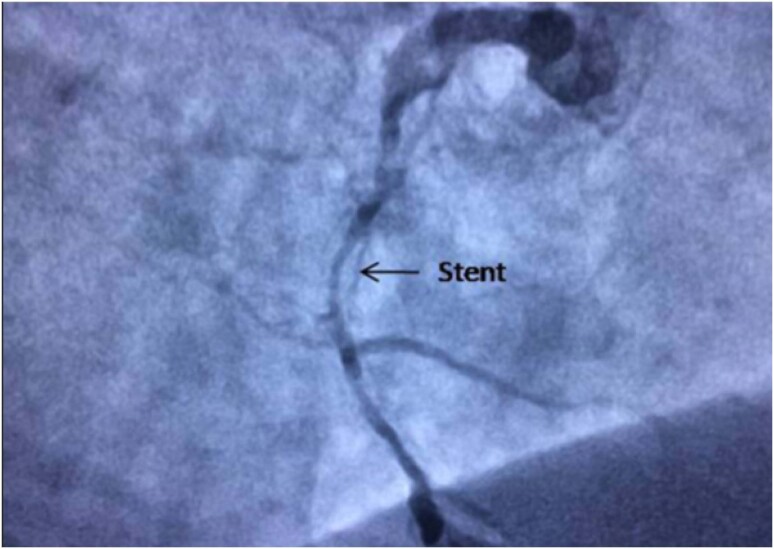
Achados angiográficos coronários com reestenose de stent na artéria coronária direita.

A análise de regressão múltipla revelou que a presença de placas ecolucentes nas artérias carótidas aumentou o risco de reestenose de *stent* coronário em 8,21 vezes (8,21; IC95% 3,58-18,82; p<0,001). No entanto, observamos que uma EIM elevada não aumentou o risco de reestenose de stent coronário (RR 1,03; IC95% 0,60-1,76; p=0,897).

## Discussão

O estudo revelou uma clara correlação entre placas ateroscleróticas ecolucentes nas artérias carótidas e reestenose de *stent* coronário avaliado 12 meses após o implante do *stent*. Pacientes com placas ecolucentes nas artérias carótidas apresentaram um risco 8,21 vezes maior de reestenose de *stent* coronário em comparação àqueles com placas ecogênicas ou nenhuma placa nas carótidas. Um estudo prévio, no entanto, relatou uma correlação entre placas ecolucentes e reestenose de *stent* coronário com um OR de 3,8.^20^ Apesar de considerados obsoletos muitas vezes, SNF foram usados em ambos os estudos, o que reflete a realidade na América Latina. Apesar de similares, os estudos diferem-se na etnicidade das populações e no segundo agente antiplaquetário empregado, uma vez que o estudo de 2008 usou ticlopidina. Uma possível justificativa para essa correlação é inflamação, comum a ambas as situações. Os macrófagos foram as primeiras células inflamatórias a serem reconhecidas como associadas à aterosclerose.^21^ Posteriormente, outros tipos de leucócitos relacionados à inflamação tais como monócitos, neutrófilos, e linfócitos foram detectados nas placas ateroscleróticas.^22^^,^^23^ As citocinas também estão relacionadas à inflamação aguda e crônica, e sua produção depende em muitos fatores rigorosamente regulados durante a inflamação. Uma ampla variedade de citocinas, tais como TNF-α, IL-1, IL-2, IL-3, IL-6, CXCL8, IL-10, IL-12, IL-15, IL-18, IFN-γ, M-CSF, TGF-β1, TGF-β2, e TGF-β3, foi detectada nas placas ateroscleróticas. Ainda, em condições de hiperlipidemia, TNF-α, IL-1, IL-6, IL-12, IL-15, e IL-18 são produzidos por macrófagos.^24^ Vários estudos sugeriram a hipótese que disfunção endotelial – causada principalmente por níveis elevados de LDL, tabagismo, hipertensão arterial, e diabetes mellitus – é a primeira etapa no desenvolvimento de aterosclerose. Assim, cada etapa da aterosclerose representaria uma diferente fase do processo inflamatório crônico.^25^

As plaquetas também exercem um importante papel no processo aterogênico. Elas podem regular a resposta imune e inflamatória pela secreção de mediadores inflamatórios que modulam o recrutamento de leucócitos aos tecidos inflamados. Plaquetas ativas, as quais expressam P-selectina, foram detectadas em diferentes fases da aterosclerose.^26^

Placas ateroscleróticas ecolucentes – diferente das placas ecogênicas, que contêm mais cálcio e tecido fibroso – são mais ricas em lipídios, elastina, e células inflamatórias, com alta concentração de macrófagos e atividade da metaoloproteinase elevada, os quais exercem importante papel na diferenciação, proliferação e migração celular, e na remodelação vascular.^27^ A presença de placa ecolucente na artéria carótida mostrou ser um preditor independente de acidente vascular cerebral e síndrome coronária aguda, incluindo infarto do miocárdio.^28^^,^^29^

A reestenose de *stent* é causada por uma combinação de fatores incluindo denudação endotelial, trauma mecânico, e desarranjo da túnica média e adventícia. Uma reação inflamatória ocorre em estruturas do *stent*, com infiltração de leucócitos, monócitos, e macrófagos. A gravidade da inflamação é diretamente proporcional ao trauma na parede arterial. Além disso, lesão mecânica da parede do vaso estimula a migração de células musculares lisas (da túnica média) e miofibroblastos (da túnica adventícia) à túnica íntima, onde elas se proliferam.^30^ A exposição das túnicas dos vasos facilita o contato com fatores da circulação sanguínea, estimulando a hiperplasia da túnica íntima. Com o passar do tempo, a celularidade diminui e a matriz extracelular começa a predominar na lesão de reestenose. Estudos histopatológicos descrevem uma relação inflamatória mais prolongada após a implantação do *stent* que após a angioplastia com balão.^31^

Kornowski et al.,^32^ relataram que a relação inflamatória da parede arterial nas artérias coronárias de suínos foi frequentemente observada um mês após o implante de *stent*. A reação inflamatória foi composta principalmente de histiócitos, linfócitos, e formação de granuloma, e de neutrófilos nas formas inflamatórias graves. Houve uma forte correlação entre a extensão da reação inflamatória e a quantidade de formação neointimal no interior dos *stents*. Segundo os estudos mencionados, que avaliaram os mecanismos de aterogênese e reestenose de *stent*, a inflamação é um link comum evidente entre placa ecolucente na artéria carótida e reestenose de *stent* coronário. Ainda, Rothwell et al.,^33^ relataram que instabilidade da placa, ou seja, com inflamação, não é meramente um fenômeno vascular local, mas ocorre simultaneamente em vários lugares no leito vascular sistêmico.

Apesar de ser um preditor de doenças cardiovasculares, uma EIM aumentada não elevou o risco para reestenose de *stent*. Tal fato é consistente com estudos prévios e com o conceito de que o tamanho da placa não contribui tanto como a instabilidade da placa para eventos cardiovasculares.^20^^,^^34^ Isso se deve possivelmente ao fato de que o espessamento da camada íntima-média da artéria carótida faz parte do processo de envelhecimento da parede arterial, e não sinônimo de aterosclerose subclínica. Contudo, alterações celulares e moleculares observadas no espessamento da íntima-média estão envolvidas no desenvolvimento e progressão de placas.^14^ Assim, um aumento na EIM sem placas concomitantes não teria relação com os processos inflamatórios na aterosclerose.

### Limitações do estudo

A validade externa deste estudo é limitada pela avaliação somente de pacientes sintomáticos, diagnosticados com angina estável. No entanto, todos os pacientes neste estudo foram submetidos à angiografia coronária, exame padrão-ouro para o diagnóstico de reestenose de *stent* coronário, o que aumenta a validade interna do estudo. Outra limitação é o fato de não termos. estudado um grupo de pacientes submetidos ao implante de SF.

## Conclusão

A presença de placa aterosclerótica ecolucente na artéria carótida representa um preditor de risco de reestenose de *stent* coronário e deveria ser considerada, juntamente com outros preditores de risco, na escolha do tipo de *stent* a ser implantado na angioplastia coronária.
