# Supplementary material for: Perfil Aterosclerótico da Artéria Carótida como Preditor de Risco para Reestenose após Implante de Stent Coronário
Source: Arq Bras Cardiol. 2021 Apr 8;116(4):727–33. [Article in Portuguese] doi: 10.36660/abc.20190650 (PMC8121410; doi:10.36660/abc.20190650)
Supplement: Translation

# Translation: Carotid Artery Atherosclerotic Profile as Risk Predictor for Restenosis After Coronary Stenting

**Authors:** Cássia da Silva Antico Rodrigues, Rodrigo Bazan, Fabrício Moreira Reis, Caroline F. S. Mazeto Pupo da Silveira, Lívia Maria Severino Hueb, Fábio Cardoso de Carvalho, Hélio Rubens de Carvalho Nunes, Katashi Okoshi, João Carlos Hueb, Silméia Garcia Zanati Bazan

**Affiliations:** 1 BotucatuSP Brazil Universidade Estadual Paulista Julio de Mesquita Filho - Faculdade de Medicina Campus de Botucatu, Botucatu, SP – Brazil

**Keywords:** Coronary Artery Disease, Atherosclerosis, Coronary restenosis, Stents, Angioplasty, Balloon, Coronary, Carotid Arteries/ultrasonography, Plaque, Atherosclerotic

## Abstract

**Background:**

The incidence of restenosis of the coronary artery after a bare-metal stent implant has been lower than in simple balloon angioplasty; however, it still shows relatively high rates.

**Objective:**

The aim of this study was to find new risk indicators for in-stent restenosis using carotid ultrasonography, that, in addition to the already existing indicators, would help in decision-making for stent selection.

**Methods:**

We carried out a cross-sectional prospective study including 121 consecutive patients with chronic coronary artery disease who had undergone percutaneous coronary intervention with repeat angiography in the previous 12 months. After all cases of in-stent restenosis were identified, patients underwent carotid ultrasonography to evaluate carotid intima-media thickness and atherosclerosis plaques. The data were analyzed by Cox multiple regression. The significance level was set a p<0.05.

**Results:**

Median age of patients was 60 years (1^st^ quartile = 55, 3^rd^ quartile = 68), and 64.5% of patients were male. Coronary angiography showed that 57 patients (47.1%) presented in-stent restenosis. Fifty-five patients (45.5%) had echolucent atherosclerotic plaques in carotid arteries and 54.5% had echogenic plaques or no plaques. Of patients with who had echolucent plaques, 90.9% presented coronary in-stent restenosis. Of those who had echogenic plaques or no plaques, 10.6% presented in-stent restenosis. The presence of echolucent plaques in carotid arteries increased the risk of coronary in-stent restenosis by 8.21 times (RR=8.21; 95%CI: 3.58-18.82; p<0.001).

**Conclusions:**

The presence of echolucent atherosclerotic plaques in carotid artery constitutes a risk predictor of coronary instent restenosis and should be considered in the selection of the type of stent to be used in coronary angioplasty.

## Introduction

The development of bare-metal stents (BMS) was a great advance in balloon angioplasty for treating symptomatic coronary artery disease. With the use of stents, restenosis can be avoided by attenuating the elastic recoil and promoting a negative geometric remodeling, resulting in reduction of vessel lumen.^1^ However, the need for new revascularizations due to in-stent restenosis was still relatively high, occurring in 10% to 20% of patients, mostly caused by excessive neointima growth, sometimes even larger than the intimal hyperplasia observed with a simple balloon angioplasty.^2^^,^^3^

More recently, drug-eluting stents (DES) were developed to reduce the high restenosis rate observed with BMS and the need for revascularization. Clinical trials have confirmed a reduction of 50-70% in the need for revascularizations of the target lesion with DES compared to BMS, although there has been no significant difference in overall mortality rate between them.^4^^-^^9^ These results have led to the preferential recommendation of DES in coronary percutaneous intervention. However, these stents are expensive and require a long period of dual antiplatelet therapy to avoid thrombosis, and hence are not recommended for all patients.^10^

In some situations such as diabetes mellitus, small vessel involvement, in-stent stent, bifurcation lesions, long or multiple lesions, and saphenous vein graft, angioplasty with stent implantation present a high risk of restenosis (30-60%). In these conditions, DES are more consistently indicated.^11^

In addition to the above-mentioned situations, little is known about the importance of atherosclerotic plaques in carotid arteries and their correlation to in-stent restenosis. This correlation is possible since inflammation is common in both cases.^12^ According to Corrado et al.,^13^ in patients undergoing coronary stent implantation, a higher frequency of in-stent restenosis is observed in those presenting greater carotid intima-media thickness (CIMT) and atherosclerotic plaques in carotid arteries.

As most risk indicators for in-stent restenosis concern coronary angiographic aspects, the objective of this study was to correlate, using ultrasonography, the carotid artery atherosclerotic profile with coronary in-stent restenosis, focusing on the presence of echolucent plaques.

## Patients and methods

### Patients

This study was approved by Botucatu Medical School Ethics Committee. All patients signed the informed consent form before participating in this study. We carried out a cross-sectional prospective study including 121 consecutive patients with chronic coronary artery disease, from February to December of 2015. All patients had undergone percutaneous coronary intervention and another angiography within 12 months. The angiographies were indicated for stable angina risk stratification, or after any confirmed myocardial ischemia in provocative tests (exercise stress test or cardiac scintigraphy with stress. Based on the directed interview we identified which patients had diabetes mellitus, dyslipidemia, or arterial hypertension. We also identified if they were tobacco users, and which medications they were taking. Coronary angiography detected previous stent implantation in coronary arteries (right coronary artery, circumflex coronary artery, anterior descending coronary artery, and their respective branches).

### Carotid Artery Duplex Ultrasonography

All procedures were performed by an experienced sonographer using a Vivid S6 echocardiograph (General Electric Medical Systems, Tirat Carmel, Israel) equipped with an 8.0 MHz frequency linear array probe. Duplex ultrasonography of the carotid artery was performed with patient in supine decubitus position. Carotid images were analyzed according to the consensus statement from the American Society of Echocardiography carotid intima-media thickness task force^14^ and the Mannheim Carotid Intima-Media Thickness Consensus,^15^ and recorded on a compact disc. Classification of CIMT by age and gender were determined based on the 75^th^ percentile of values proposed in the CAPS study.^16^

CIMT was measured using a double-line pattern visualized by echotomography on both common carotid arteries in a longitudinal image. This double-line pattern comprises the leading edges of the lumen–intima and media–adventitia interfaces. Mean values were calculated on a 10 mm segment next to the posterior wall of carotid bulb. Plaque was considered a focal structure when it encroached into the arterial lumen by at least 0.5 mm, or corresponded to 50% of surrounding CIMT value, or demonstrated a thickness of >1.5 mm, as measured from the media–adventitia to lumen–intima interface. Plaques were described according to the classification by Gray-Weale et al.^17^ In brief, type I plaque is uniformly echolucent; type II is predominantly echolucent; type III is predominantly echogenic; and type IV is uniformly echogenic. For statistical analysis, types I and II were called echolucent and types III and IV echogenic.

### Coronary angiography

Coronary angiographies were performed by transradial cardiac catheterization. After selective coronary angiography and stent identification, restenosis was evaluated by quantitative angiography. In-stent restenosis was defined as a lumen reduction of 50% or greater.^18^^,^^19^

### Statistical analysis

Continuous variables were presented as medians and minimum and maximum values. Categorical variables were expressed as absolute values or frequency (%). The analysis of predictors of risk for in-stent restenosis at 12 months of follow-up was performed in two stages. In step 1, individual relative risk for each potential predictor was estimated. Then in phase 2, the model of multiple Cox regression was adjusted for the risk of in-stent restenosis with the predictors most strongly associated (p <0.05) with restenosis detected in phase 1. Values of p <0.05 were considered as statistically significant. All statistical analyses were performed using SPSS v21.0 software.

## Results

The median age of the 121 patients was 60 years (1^st^ quartile = 55, 3^rd^ quartile = 68); 78 patients (64.5%) were male. Fifty-eight (47.9%) patients were smokers, 47 (38.8%) were diabetic, 91 (75.2%) had systemic hypertension, and 119 (98.3%) had dyslipidemia. After adjusting the Cox multiple-regression model for the risk of in-stent restenosis by potential predictors of restenosis, we observed that there was no statistically significant difference in distribution of these variables in the subgroups with or without in-stent restenosis (Table 1).

**Table 1:** In-stent restenosis risk estimated for each variable

Variable		RR	95%CI	p
Age		0.99	0.96-1.02	0.555
Males		1.87	1.01-3.46	0.048
**Medical history**				
	AH	0.85	0.47-1.51	0.567
	DM	1.07	0.63-1.81	0.815
	Tobacco use	1.21	0.72-2.03	0.478
	Dyslipidemia	0.94	0.13-6.80	0.952
**Carotid artery US**				
	Echolucent plaques	8.57	3.89-18.90	<0.001
	CIMT (increased)	1.88	1.11-3.15	0.017
**Coronary with stent**				
	LAD	1.23	0.72-2.07	0.450
	RCA	0.76	0.44-1.32	0.330
	LCX	0.85	0.45-1.60	0.607

AH: arterial hypertension; DM: diabetes mellitus; US: ultrasonography; CIMT: carotid intima-media thickness; LAD: left anterior descending artery; RCA: right coronary artery; LCX: left circumflex artery.

Stent locations were as follow: left anterior descending artery (LAD) in 50 patients (41.3%), right coronary artery (RCA) in 34 patients (28.1%), left circumflex artery (LCX) in 19 patients (15.7%), both LAD and RCA in 9 patients (7.4%), both LAD and LCX in 5 patients (4.1%), and both RCA and LCX in 4 patients (3.3%). Angiographies showed that 57 patients (47.1%) presented coronary in-stent restenosis, and the stent location did not influence in-stent restenosis rates (Table 1).

Most patients were taking aspirin (97.5%), statin (92.6%), angiotensin converting enzyme inhibitors or angiotensin receptor blockers (80.2%), beta-blockers (88.4%), and 27.3% were taking clopidogrel.

Fifty-five patients (45.5%) showed echolucent plaques in carotid arteries and 66 patients (54.5%) presented echogenic plaques or no plaques. Fifty patients (90.9%) with echolucent plaques and only seven (10.6%) of those with echogenic plaques or no plaques showed in-stent restenosis (Figure 1).

**Figure 1:** Percentage of patients with coronary in-stent restenosis in the subgroups of patients with echolucent plaques and patients with echogenic/no plaque in carotid arteries.

Ultrasonography images of carotid plaques and coronary angiographic findings are shown in Figures 2 and 3, respectively.

**Figure 2:** Ultrasound images of type II atherosclerotic plaque in the left carotid artery.

**Figure 3:** Coronary angiographic findings with stent restenosis in the right coronary artery.

The analysis of multiple regression revealed that the presence of echolucent plaques in carotid arteries increased the risk of coronary in-stent restenosis by 8.21 times (RR 8.21; 95%CI; 3.58-18.82; p<0.001). However, we observed that increased CIMT did not increase the risk of coronary in-stent restenosis (RR 1.03; 95%CI 0.60-1.76; p=0.897).

## Discussion

This study revealed a clear correlation between echolucent atherosclerotic plaques in carotid arteries and coronary in-stent restenosis evaluated at 12 months after stent implantation. Patients with echolucent plaques in carotid arteries presented an 8.21 times greater risk of coronary in-stent restenosis than those with echogenic plaques or no atherosclerotic plaques in carotid. A previous study, however, reported a correlation between echolucent plaques and coronary in-stent restenosis with an OR of 3.8.^20^ Although often considered obsolete, BMS were used in both studies, which reflects most appropriately the current reality in Latin America. Though similar, the studies differ as to ethnicity and the second antiplatelet agent employed, as the 2008 study used ticlopidine. A possible justification for this correlation is an inflammatory state, which is common in both situations. Macrophages were the first inflammatory cells to be recognized to be associated with atherosclerosis.^21^ Later, other types of inflammation-related leukocytes such as monocytes, neutrophils, and lymphocytes were detected in atherosclerotic plaques.^22^^,^^23^ Cytokines are also related to acute and chronic inflammation, and their production depends on many strictly regulated factors during inflammation. A wide range of cytokines, such as TNF-α, IL-1, IL-2, IL-3, IL-6, CXCL8, IL-10, IL-12, IL-15, IL-18, IFN-γ, M-CSF, TGF-β1, TGF-β2, and TGF-β3 have been found in atherosclerotic plaques. Furthermore, under hyperlipidemic conditions TNF-α, IL-1, IL-6, IL-12, IL-15, and IL-18 are produced by macrophages.^24^ Several studies have suggested the hypothesis that endothelial dysfunction — mostly caused by elevated LDL, tobacco, arterial hypertension, and diabetes mellitus — is the first step towards atherosclerosis. Therefore, each step of atherosclerosis would represent a different phase of the chronic inflammatory process.^25^

Platelets also play an important role in the atherogenic process. They can regulate immune and inflammatory responses by secreting inflammatory mediators that modulate leukocyte recruitment to the inflamed tissues. Activated platelets, which express P-selectin, have been detected in different phases of atherosclerosis.^26^

Echolucent atherosclerotic plaques — unlike echogenic plaques, which contain more calcium and fibrous tissue — are much richer in lipids, elastin, and inflammatory cells, with high macrophage concentration and metalloproteinase activity, which plays an important role in cellular differentiation, proliferation and migration, and also in vascular remodeling.^27^ Echolucent plaque in carotid artery has been shown to be an independent predictor of stroke and acute coronary syndrome, including myocardial infarction.^28^^,^^29^

In-stent restenosis is caused by a combination of factors including endothelial denudation, mechanical trauma, and derangement of the tunica media and adventitia. An inflammatory reaction occurs in the stent structures, with leukocyte, monocyte, and macrophage infiltration; inflammation severity is directly proportional to arterial wall trauma. Mechanical injury of the vessel wall stimulates the migration of smooth muscle cells (from the tunica media) and myofibroblasts (from the tunica adventitia) to the tunica intima, where they proliferate.^30^ Exposure of the vessel tunics facilitates contact with blood circulating factors, stimulating intima tunic hyperplasia. As time passes, cellularity decreases and the extracellular matrix begins to predominate in the restenosis lesion. Histopathological studies describe a more prolonged inflammatory reaction after stent implantation than after balloon angioplasty.^31^

Kornowski et al.^32^ reported that inflammatory reaction of arterial wall in porcine coronary arteries was frequently observed 1 month after stent implantation. The inflammatory reaction was mainly composed of histiocytes, lymphocytes, and granuloma formation, and also neutrophils in the most severe inflammatory forms. There was a strong correlation between the extent of inflammatory reaction and the amount of neointimal formation within the stents. According to the above studies that evaluated the mechanisms of atherogenesis and in-stent restenosis, inflammation is an evident common link between echolucent plaque in carotid artery and coronary in-stent restenosis. Furthermore, Rothwell et al.^33^ reported that plaque instability, i.e., with inflammation, is not a merely local vascular phenomenon, but occurs simultaneously at multiple sites in the systemic vascular bed.

Despite being a predictor of cardiovascular diseases, increased CIMT did not elevate the risk of in-stent restenosis. This is consistent with previous studies and with the concept that plaque size does not contribute as much as plaque instability to cardiovascular events.^20^^,^^34^ This was possibly because carotid intima-media thickening is part of the arterial wall aging process, and not synonymous of subclinical atherosclerosis. However, cellular and molecular changes observed in intima-media thickening have been implicated in plaque development and progression.^14^ Thus, an increase in CIMT with no concomitant plaques would have no relation to the inflammatory processes in atherosclerosis.

### Study limitations

The external validity of this study is limited due to the evaluation of symptomatic patients diagnosed with stable angina only. However, the fact that all patients in this study underwent coronary angiography, which is the gold standard exam for the diagnosis of coronary stent restenosis, increases its internal validity. Another limitation found was that we did not study a group of patients submitted to DES.

## Conclusion

The presence of echolucent atherosclerotic plaque in carotid artery represents a risk predictor of coronary in-stent restenosis and should be considered along with other risk predictors in the decision-making on the type of stent to be implanted in coronary angioplasty.
